# Successful management of Still’s disease with severe liver injury through the administration of baricitinib: a case report

**DOI:** 10.3389/fmed.2025.1591431

**Published:** 2025-07-28

**Authors:** Dabin Tang, Yubao Jiang, Yanying Zhang, Jingjing Xie, Jianyong Zhang

**Affiliations:** ^1^The Fourth Clinical Medical College of Guangzhou University of Chinese Medicine, Shenzhen, Guangdong, China; ^2^Department of Rheumatology, Shenzhen Traditional Chinese Medicine Hospital, Shenzhen, Guangdong, China

**Keywords:** Still’s disease, glucocorticoid-dependent, severe liver injury, baricitinib, adult-onset Still’s disease

## Abstract

Still’s disease (SD) is a rare systemic autoinflammatory disorder of unknown etiology, clinically characterized by a triad of high-spiking fevers, an evanescent salmon-colored rash, and arthritis. The disease exhibits considerable heterogeneity, ranging from mild manifestations to severe, life-threatening complications such as macrophage activation syndrome (MAS). Management strategies are tailored according to disease severity, with glucocorticoids remaining the mainstay of treatment for moderate to severe cases. However, a subset of patients becomes glucocorticoid-dependent or refractory, necessitating the use of steroid-sparing immunosuppressive agents. Recent advancements in biologic therapies have significantly improved disease outcomes and are increasingly adopted as first-line therapies. The Janus kinase-signal transducer and activator of transcription (JAK–STAT) signaling pathway, known to be activated in various autoimmune and inflammatory conditions, has emerged as a promising therapeutic target. Herein, we present a case of SD complicated by severe liver injury, which was successfully managed with baricitinib following the failure of conventional synthetic disease-modifying antirheumatic drugs.

## Introduction

Still’s disease (SD) is a rare, systemic inflammatory disorder of unknown origin, marked by recurrent high fevers, a transient salmon-pink rash, and inflammatory arthritis. SD has a historical context in which it has been recognized under two distinct denominations, differentiated primarily by the age of onset: systemic juvenile idiopathic arthritis (sJIA) and adult-onset Still’s disease (AOSD). The demarcation between these two entities is based on an arbitrary age threshold of 16 years. Nevertheless, compelling evidence indicates that sJIA and AOSD correspond to the same rare, non-familial (sporadic) systemic inflammatory disorder, manifesting at different stages of the lifespan (1, 2). As a diagnosis of exclusion, it requires careful differentiation from infectious, neoplastic, and other autoimmune conditions. Beyond its primary symptoms, SD can involve multiple organ systems, leading to complications such as serositis, lymph node enlargement, hepatosplenomegaly, and hematologic abnormalities. In severe cases, patients may experience life-threatening conditions, including macrophage activation syndrome (MAS), disseminated intravascular coagulation (DIC), and acute hepatic failure, all of which critically influence prognosis and therapeutic decisions (3).

Hepatic involvement is a frequent finding in SD, with mild to moderate liver enzyme elevation being common (4). However, severe hepatic dysfunction is rare, occurring in cases of fulminant disease or MAS. The underlying mechanism of liver injury is thought to be driven by inflammatory cytokines, including interleukin (IL)-1, IL-6, IL18, tumor necrosis factor (TNF)-*α* and ferritin, leading to systemic inflammation and tissue damage (5–9). Treatment selection must balance efficacy with safety, especially in patients with hepatic dysfunction.

Therapeutic approaches for SD are guided by disease severity. Non-steroidal antiinflammatory drugs (NSAIDs) are typically employed in mild cases, while glucocorticoids remain the cornerstone of treatment for more severe presentations. In patients who develop glucocorticoid dependence or refractory disease, steroidsparing immunosuppressive agents, such as cyclosporine, are commonly utilized. The introduction of biologic therapies targeting interleukin (IL)-1 and IL-6 has significantly improved clinical outcomes and transformed the therapeutic landscape of SD (2, 6, 10). Baricitinib, a Janus Kinase (JAK) inhibitor, modulates immune responses by blocking the signal transducer and activator of transcription (STAT) pathway, reducing inflammatory cytokine overproduction. It offers a broad anti-inflammatory effect with minimal hepatotoxicity (11), making it a viable alternative for SD patients with liver injury.

This report presents a case of SD complicated by severe hepatic dysfunction, successfully treated with baricitinib following the failure of high-dose glucocorticoids and csDMARDs. This case highlights the potential role of JAK inhibitors in SD management, particularly in patients unresponsive to biologics and requiring a treatment option with minimal hepatic toxicity.

## Case presentation

A 20-year-old woman with a history of sJIA and MAS diagnosed 8 years prior presented with a three-month history of recurrent fever (up to 38.5°C) and polyarticular arthritis. NSAIDs treatment led to symptom resolution, but 6 days before admission, she experienced fever (up to 39.2°C), evanescent rash, pharyngitis, cervical lymphadenopathy, myalgia, and arthritis.

**Figure 1:**
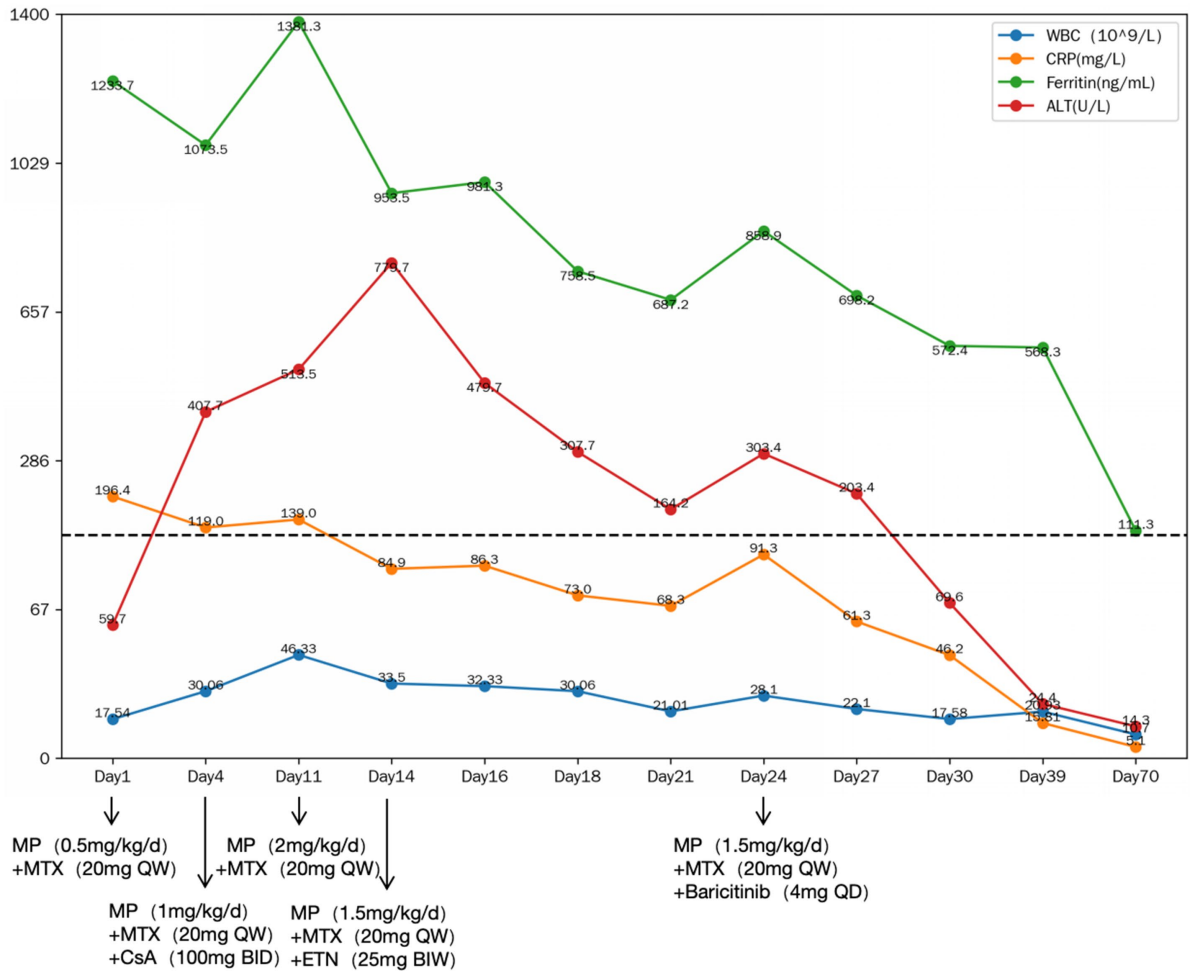
The medical records during the treatment. MP, methylprednisolone; MTX, methotrexate; CsA, cyclosporine A; ETN, etanercept; WBC, white blood cell; CRP, C-reactive protein; ALT, alanine aminotransferase; QD, once daily; BID, (twice daily); QW, once weekly; BIW, twice weekly.

Laboratory findings on admission included leukocytosis 17.54 × 10^9/L (normal range: 3.50–9.50^9/L), neutrophilia 14.99 × 10^9/L (normal range: 1.80–6.30^9/L), hemoglobin 102 g/L (normal range: 115–150 g/L), C-reactive protein (CRP) 196.4 mg/L (normal range: 0.0–6.0 mg/L), alanine aminotransferase (ALT) 59.7 U/L (normal range: 7.0–40.0 U/L), aspartate aminotransferase (AST) 24.2 U/L (normal range: 13.0–35.0 U/L), gamma-glutamyl transpeptidase (*γ*-GGT) 166.5 U/L (normal range: 7.0–45.0 U/L), total bilirubin (TBIL) 32.1 μmol/L (normal range: 2.0–23.0 μmol/L), direct bilirubin (DBIL) 22.7 μmol/L (normal range: 0.0–6.8 μmol/L), alkaline phosphatase (ALP) 84 U/L (normal range: 35.0–100.0 U/L), prothrombin time (PT) 13.6 s (normal range: 11.0–14.5 s), activated partial thromboplastin time (APTT) 41.1 s (normal range: 28.0–43.0 s), erythrocyte sedimentation rate (ESR) 90 mm/h (normal range: 0.0–20.0 mm/h), ferritin 1233.7 ng/mL (normal range: 11.0–306.8 ng/mL), and IL-6 70.69 pg./mL (normal range: 0.0–10.3 pg./mL). Infectious, malignant, and other autoimmune disease workups were negative. SD was diagnosed based on Yamaguchi criteria (12). NSAIDs were ineffective, necessitating methotrexate (20 mg weekly) and glucocorticoid (0.5 mg/kg/day) initiation. Despite treatment, fever, rash, and arthritis recurred within 3 days, accompanied by worsening leukocytosis (30.06 × 10^9/L), ALT (407.7 U/L), *γ*-GGT (470.7 U/L), TBIL 61.7 μmol/L, DBIL 60.0 μmol/L, ALP 154 U/L, PT 19.9 s, APTT 48.2 s. Cyclosporine (100 mg twice daily) was added, and glucocorticoid increased to 1 mg/kg/day, but symptoms recurred within 7 days. Further laboratory evaluation showed worsening leukocytosis (46.33 × 10^9/L), ALT (513.5 U/L), and *γ*-GGT (353.9 U/L), TBIL 76.1 μmol/L, DBIL 74.1 μmol/L, ALP 165 U/L, PT 16.5 s, APTT 51.4 s, necessitating glucocorticoid escalation to 2 mg/kg/day, which improved symptoms but worsened liver enzymes (ALT 779.7 U/L, AST 292.9 U/L, *γ*-GGT 604.2 U/L). Drug-induced hepatotoxicity was suspected, leading to cyclosporine discontinuation (13). Glucocorticoid tapering (1.5 mg/kg/day) led to arthritis recurrence. Etanercept was ineffective, prompting a switch to baricitinib (4 mg/day), resulting in gradual improvement and glucocorticoid tapering (10 mg/week) until cessation without relapse. Baricitinib was discontinued after 6 months, with the patient remaining in remission on methotrexate at a dosage of 10 mg per week for 3 years. The medical records documenting the treatment course are presented in Figure 1.

## Discussion and conclusion

This case report highlights the complex interplay between SD and hepatic dysfunction, underscoring the challenges of disease management in cases. SD has historically been classified into two distinct entities based primarily on the age of onset: sJIA and AOSD. This distinction relies on an arbitrary age threshold of 16 years. However, growing evidence supports the notion that sJIA and AOSD represent the same rare, sporadic systemic inflammatory disorder manifesting at different stages of life. Both conditions are characterized by dysregulated innate immune activation with autoinflammatory features in the early phase, followed by aberrant adaptive immune responses in the chronic phase. Clinically, sJIA and AOSD share four hallmark features: high-spiking fevers, arthralgia or arthritis, a salmon-pink macular or maculopapular rash, and elevated leukocyte and neutrophil counts. They also present with similar additional manifestations, including hepatomegaly, splenomegaly, lymphadenopathy, and serositis, as well as common laboratory abnormalities such as elevated ESR, CRP, and hyperferritinemia. The disease course in both entities may follow monocyclic, polycyclic, or chronic patterns and often demonstrates a dichotomous phenotype, with patients exhibiting either predominantly systemic inflammatory or chronic articular features. Moreover, both sJIA and AOSD carry a risk of serious, potentially life-threatening complications, including MAS, hepatitis, and interstitial lung disease which necessitate timely recognition and intervention to improve outcomes (1, 2).

Hepatic involvement is a well-recognized but variable feature of SD, occurring in approximately 43–76% of cases (14). While most patients experience mild to moderate liver enzyme elevations, a small subset progresses to severe hepatic dysfunction, fulminant hepatitis, or liver failure. The underlying mechanism of liver injury in SD remains unclear but is believed to be driven by a cytokine-mediated inflammatory response, leading to hepatocellular injury, cholestasis, and, in extreme cases, liver failure (4). Given this risk, treatment strategies must carefully balance efficacy and safety to prevent hepatic complications. In the present case, the patient had a history of sJIA and MAS 8 years ago, and the current episode represents a recurrence of the disease. Timely diagnosis and treatment are of great importance to avoid the development of life-threatening complications due to delayed intervention, such as MAS. Standard treatment with high-dose glucocorticosteroids (2 mg/kg/day) was effective, however, long-term high-dose corticosteroids use poses significant adverse effects, rendering necessary the use of steroid-sparing agents to achieve disease control while minimizing toxicity (2).

One of the critical challenges in this case was differentiating between SD-related hepatic dysfunction and potential drug-induced liver injury (DILI). Liver dysfunction in SD can result from disease-related inflammation, MAS, or medication side effects (15). Accurately identifying the primary cause of hepatic injury is crucial to guiding treatment decisions and avoiding premature discontinuation of effective therapies. While glucocorticosteroids are not traditionally associated with hepatotoxicity, emerging evidence suggests they may contribute indirectly through metabolic dysregulation, promoting hepatic steatosis or exacerbating pre-existing liver disease (16). Rare cases of idiosyncratic DILI due to glucocorticosteroids have been reported, generally presenting with transient liver enzyme elevations that resolve upon dose reduction (17, 18).

Methotrexate and cyclosporine, two widely used immunosuppressive agents, also pose risks of hepatotoxicity. Methotrexate-induced liver injury is typically cumulative and dose-dependent, manifesting as elevated transaminases, hepatic steatosis, fibrosis, and, in severe cases, cirrhosis (19, 20). Cyclosporine-induced liver toxicity, in contrast, is often cholestatic and characterized by elevated bilirubin, alkaline phosphatase, and *γ*-GGT. Given these risks, careful monitoring of liver function is essential when using these agents, particularly in patients with pre-existing hepatic impairment (13, 21). In this case, drug-induced liver injury could not be entirely ruled out. However, the absence of a clear temporal relationship between medication exposure and enzyme elevations, along with persistent hepatic dysfunction despite glucocorticosteroid therapy, strongly suggested that SD itself was the primary driver of liver injury. This aligns with previous reports that severe hepatic dysfunction in SD is predominantly cytokine-mediated rather than drug-induced.

In light of the patient’s clinical trajectory, including progressive hepatic dysfunction and reliance on high-dose corticosteroids, the initiation of an alternative therapeutic approach was warranted. Moreover, an expanding body of evidence supports the use of biologic agents targeting IL-1 and IL-6, which have markedly improved clinical outcomes and are now recognized as first-line therapeutic options in the management of SD (2, 22). Baricitinib, a selective JAK inhibitor, was chosen for its ability to target multiple inflammatory pathways simultaneously. JAK inhibitors block the JAKSTAT signaling pathway, disrupting key cytokines such as IL-6, IFN-*γ*, and TNF-*α*, all implicated in SD pathogenesis. While data on the use of JAK inhibitors in SD remain limited, emerging evidence indicates that they may offer therapeutic benefit, particularly in patients refractory to conventional treatments. Specifically, baricitinib has demonstrated efficacy in managing SD, with some patients achieving partial or complete remission, and exhibiting a notable corticosteroid-sparing effect-an outcome of particular relevance in difficult-to-treat cases that have failed to respond to conventional or biologic therapies (23, 24). Notably, baricitinib has a relatively low risk of hepatotoxicity, making it an attractive option for patients with liver dysfunction (11).

Following the initiation of baricitinib, the patient demonstrated rapid and sustained disease control, with resolution of systemic symptoms, fever, and arthritis. Importantly, liver enzyme levels normalized significantly, suggesting that baricitinib not only controlled systemic inflammation but also improved SD-related hepatic dysfunction. Additionally, baricitinib facilitated successful glucocorticoid tapering, reducing the long-term risks associated with prolonged high-dose steroid use.

Despite the positive outcome, this case has several limitations. First, the patient’s history of sJIA and MAS may have influenced disease course and treatment response. Earlier administration of high-dose corticosteroids might have led to faster disease control. Second, IL-6 and IL-1 inhibitors, commonly used in SD, were not trialed in this case because of unavailability. The decision to use baricitinib instead was based on clinical judgment, but comparative efficacy studies between JAK inhibitors and IL-1/IL-6 inhibitors in SD remain lacking. Third, while clinical findings suggested SD-related hepatic dysfunction, additional diagnostic tools such as liver ultrasound or biopsy could have provided definitive insights into the etiology of liver impairment. Future studies should focus on refining diagnostic approaches to distinguish between SD-related hepatic injury and drug-induced hepatotoxicity.

In conclusion, this case illustrates the successful use of baricitinib in SD complicated by severe hepatic dysfunction. Baricitinib effectively controlled systemic inflammation, normalized liver function, and enabled glucocorticoid tapering. Given the absence of standardized treatment guidelines for SD with hepatic involvement, JAK inhibitors may represent a valuable therapeutic option, particularly for patients with steroid-dependent disease. Further research is necessary to evaluate the longterm efficacy, safety, and optimal role of JAK inhibitors in managing SD, but this case contributes to growing evidence supporting their utility in complex cases.

## Data Availability

The raw data supporting the conclusions of this article will be made available by the authors, without undue reservation.

## References

[1] De Matteis A Bindoli S De Benedetti F Carmona L Fautrel B Mitrovic S. Systemic juvenile idiopathic arthritis and adult-onset Still's disease are the same disease: evidence from systematic reviews and Meta-analyses informing the 2023 EULAR/PReS recommendations for the diagnosis and management of Still's disease. Ann Rheum Dis. (2024) 83:1748–61. doi: 10.1136/ard-2024-225853, PMID: 39317414 PMC11671913

[2] Fautrel B Mitrovic S De Matteis A Bindoli S Anton J Belot A. EULAR/PReS recommendations for the diagnosis and Management of Still's disease, comprising systemic juvenile idiopathic arthritis and adult-onset Still's disease. Ann Rheum Dis. (2024) 83:1614–27. doi: 10.1136/ard-2024-225851, PMID: 39317417 PMC11672000

[3] Ruscitti P Cantarini L Nigrovic PA McGonagle D Giacomelli R. Recent advances and evolving concepts in Still's disease. Nat Rev Rheumatol. (2024) 20:116–32. doi: 10.1038/s41584-023-01065-6, PMID: 38212542

[4] Feist E Mitrovic S Fautrel B. Mechanisms, biomarkers and targets for adult-onset Still's disease. Nat Rev Rheumatol. (2018) 14:603–18. doi: 10.1038/s41584-018-0081-x, PMID: 30218025 PMC7097309

[5] Church LD Cook GP McDermott MF. Primer: inflammasomes and interleukin-1 beta in inflammatory disorders. Nat Clin Pract Rheumatol. (2008) 4:34–42. doi: 10.1038/ncprheum0681, PMID: 18172447

[6] Ortiz-Sanjuán F Blanco R Calvo-Rio V Narvaez J Rubio Romero E Olivé A. Efficacy of tocilizumab in conventional treatment-refractory adult-onset Still's disease: multicenter retrospective open-label study of thirty-four patients. Arthritis Rheumatol. (2014) 66:1659–65. doi: 10.1002/art.38398, PMID: 24515813

[7] Priori R Barone F Alessandri C Colafrancesco S McInnes IB Pitzalis C. Markedly increased IL-18 liver expression in adult-onset Still's disease-related hepatitis. Rheumatology (Oxford). (2011) 50:776–80. doi: 10.1093/rheumatology/keq397, PMID: 21149398

[8] Kraetsch HG Antoni C Kalden JR Manger B. Successful treatment of a small cohort of patients with adult onset of Still's disease with infliximab: first experiences. Ann Rheum Dis. (2001) 60:iii55–7. doi: 10.1136/ard.60.90003.iii55 11890655 PMC1766667

[9] Jia J Wang M Meng J Ma Y Wang Y Miao N. Ferritin triggers neutrophil extracellular trap-mediated cytokine storm through MSR 1 contributing to adult-onset Still's disease pathogenesis. Nat Commun. (2022) 13:6804. doi: 10.1038/s41467-022-34560-7, PMID: 36357401 PMC9648446

[10] Henderson C Wilson M Pham T-H Dolan G Gobbo A Snyder C. Safety and efficacy of Il-1 trap in resistant adult onset Still’s disease: 24 month follow-up of open label treatment and biomarkers of response. Arthritis Rheum. (2010) 62:S765.

[11] Genovese MC Kremer J Zamani O Ludivico C Krogulec M Xie L. Baricitinib in patients with refractory rheumatoid arthritis. N Engl J Med. (2016) 374:1243–52. doi: 10.1056/NEJMoa1507247, PMID: 27028914

[12] Yamaguchi M Ohta A Tsunematsu T Kasukawa R Mizushima Y Kashiwagi H. Preliminary criteria for classification of adult Still’s disease. J Rheumatol. (1992) 19:424–30. 1578458

[13] Bohme M Buchler M Muller M Keppler D. Differential inhibition by cyclosporins of primary-active ATP-dependent transporters in the hepatocyte canalicular membrane. FEBS Lett. (1993) 333:193–6. doi: 10.1016/0014-5793(93)80403-h, PMID: 8224162

[14] Zhu G Liu G Liu Y Xie Q Shi G. Liver abnormalities in adult onset Still's disease: a retrospective study of 77 Chinese patients. J Clin Rheumatol. (2009) 15:284–8. doi: 10.1097/RHU.0b013e3181b57199, PMID: 19734733

[15] Hoofnagle JH Bjornsson ES. Drug-induced liver injury-types and phenotypes. N Engl J Med. (2019) 381:264–73. doi: 10.1056/NEJMra1816149, PMID: 31314970

[16] Bjornsson ES Vucic V Stirnimann G Robles-Diaz M. Role of corticosteroids in drug-induced liver injury. A systematic review. Front Pharmacol. (2022) 13:820724. doi: 10.3389/fphar.2022.820724, PMID: 35222034 PMC8867035

[17] Zoubek ME Pinazo-Bandera J Ortega-Alonso A Hernandez N Crespo J Contreras F. Liver injury after methylprednisolone pulses: a disputable cause of hepatotoxicity. A case series and literature review. United European. Gastroenterol J. (2019) 7:825–37. doi: 10.1177/2050640619840147, PMID: 31316787 PMC6620870

[18] Kajiwara A Kawamura Y Kinowaki K Muraishi N Iritani S Akuta N. A case of drug-induced acute liver failure caused by corticosteroids. Clin J Gastroenterol. (2022) 15:946–52. doi: 10.1007/s12328-022-01661-1, PMID: 35913606

[19] O'Connor GT Olmstead EM Zug K Baughman RD Beck JR Dunn JL. Detection of hepatotoxicity associated with methotrexate therapy for psoriasis. Arch Dermatol. (1989) 125:1209–17. doi: 10.1001/archderm.1989.01670210047006, PMID: 2774596

[20] Newman M Auerbach R Feiner H Holzman RS Shupack J Migdal P. The role of liver biopsies in psoriatic patients receiving long-term methotrexate treatment. Improvement in liver abnormalities after cessation of treatment. Arch Dermatol. (1989) 125:1218–24. doi: 10.1001/archderm.1989.01670210056007 2774597

[21] Kadmon M Klünemann C Böhme M Ishikawa T Gorgas K Otto G. Inhibition by cyclosporin a of adenosine triphosphate-dependent transport from the hepatocyte into bile. Gastroenterology. (1993) 104:1507–14. doi: 10.1016/0016-5085(93)90363-h 7683296

[22] Bindoli S De Matteis A Mitrovic S Fautrel B Carmona L De Benedetti F. Efficacy and safety of therapies for still’s disease and macrophage activation syndrome (Mas): a systematic review informing the eular/pres guidelines for the management of still’s disease. Ann Rheum Dis. (2024) 83:1731–47. doi: 10.1136/ard-2024-225854 39317415 PMC11671904

[23] Ladhari C Jorgensen C Pers YM. Treatment of refractory adult onset Still's disease with combination Anakinra and Baricitinib therapy. Rheumatology (Oxford). (2019) 58:736–7. doi: 10.1093/rheumatology/key414, PMID: 30590753

[24] Gillard L Pouchot J Cohen-Aubart F Kone-Paut I Mouterde G Michaud M. Jak inhibitors in difficult-to-treat adult-onset Still's disease and systemic-onset juvenile idiopathic arthritis. Rheumatology (Oxford). (2023) 62:1594–604. doi: 10.1093/rheumatology/keac440, PMID: 35920788

